# Synapse Type-Dependent Expression of Calcium-Permeable AMPA Receptors

**DOI:** 10.3389/fnsyn.2018.00034

**Published:** 2018-10-12

**Authors:** Txomin Lalanne, Julia Oyrer, Mark Farrant, P. Jesper Sjöström

**Affiliations:** ^1^Department of Biomedicine, Institute of Physiology, University of Basel, Basel, Switzerland; ^2^The Florey Institute of Neuroscience and Mental Health, University of Melbourne, Parkville, VIC, Australia; ^3^Department of Neuroscience, Physiology and Pharmacology, University College London, London, United Kingdom; ^4^Centre for Research in Neuroscience, Department of Neurology and Neurosurgery, Brain Repair and Integrative Neuroscience Program, Montreal General Hospital, The Research Institute of the McGill University Health Centre, Montreal, QC, Canada

**Keywords:** AMPAR, NMDAR, neocortex, hippocampus, parvalbumin, somatostatin, interneuron, synapse-type-specific plasticity

## Abstract

Calcium-permeable (CP) AMPA-type glutamate receptors (AMPARs) are known to mediate synaptic plasticity in several different interneuron (IN) types. Recent evidence suggests that CP-AMPARs are synapse-specifically expressed at excitatory connections onto a subset of IN types in hippocampus and neocortex. For example, CP-AMPARs are found at connections from pyramidal cells (PCs) to basket cells (BCs), but not to Martinotti cells (MCs). This synapse type-specific expression of CP-AMPARs suggests that synaptic dynamics as well as learning rules are differentially implemented in local circuits and has important implications not just in health but also in disease states such as epilepsy.

## Introduction

AMPA-type glutamate receptors (AMPARs) are ligand-gated ion channels that mediate fast excitatory transmission throughout the CNS. They exist as homomeric or heteromeric assemblies of the pore-forming subunits GluA1, -2, -3, and -4, encoded by the genes, *GRIA1-4* (Traynelis et al., 2010). Receptor heterogeneity is increased by RNA processing events, alternative splicing and adenosine-to-inosine (A-to-I) RNA editing (Penn and Greger, 2009), and by the association of the various AMPARs with different members of large pool of auxiliary proteins (Schwenk et al., 2009; von Engelhardt et al., 2010; Schwenk et al., 2012; Shanks et al., 2012). Importantly, differences in the core subunit and auxiliary protein content, as well as a variety of post-translational modifications, can considerably alter the trafficking and functional properties of the receptors (Traynelis et al., 2010; Jackson and Nicoll, 2011; Greger et al., 2017).

Although patterns of subunit expression and different inter-subunit affinities dictate that the majority of AMPARs in the brain are heteromeric assemblies containing GluA2, receptors lacking GluA2 also exist (Wenthold et al., 1996; Sans et al., 2003; Lu et al., 2009; Rozov et al., 2012). The GluA2 subunit plays a particularly significant role in dictating AMPAR ion selectivity and voltage dependence. RNA editing of GluA2 replaces a genomically encoded glutamine with a positively charged arginine at the Q/R site in the M2 re-entrant transmembrane loop that forms the lining of the channel pore. The positively charged arginine prevents the passage of Ca^2+^ ions (Hume et al., 1991; Sommer et al., 1991; Burnashev et al., 1992), thus AMPARs containing edited GluA2 are termed Ca^2+^ impermeable (CI). Conversely, the absence of GluA2, or lack of editing, gives rise to a subset of AMPARs that are Ca^2+^ permeable (CP) (Jonas et al., 1994; Brusa et al., 1995). CP-AMPARs exhibit voltage-dependent channel block by endogenous intracellular polyamines such as spermine, which greatly limits current flow at depolarized voltages (Bowie and Mayer, 1995; Donevan and Rogawski, 1995; Kamboj et al., 1995; Koh et al., 1995a) and thus intracellular spermine-dependent rectification is an oft-used proxy for Ca^2+^ permeability (but see Bowie, 2012). GluA2-lacking (CP-) AMPARs typically desensitize faster than GluA2-containing (CI-) AMPARs (Geiger et al., 1995; Angulo et al., 1997; Sobolevsky, 2015) and have a higher single-channel conductance (Swanson et al., 1997; Feldmeyer et al., 1999).

While less abundant than their CI counterparts, CP-AMPARs are nevertheless widespread and have often been observed at excitatory connections onto inhibitory neurons (INs), where they play several important roles as discussed below (Hestrin, 1993; Mahanty and Sah, 1998; Kullmann and Lamsa, 2007; Oren et al., 2009). However, it remains unclear whether CP-AMPAR expression is restricted to specific IN subtypes (Zeisel et al., 2015; Akgul and McBain, 2016; Tasic et al., 2016) or is rather a reflection of developmental origin (Matta et al., 2013). Here we review recent studies indicating cell-type-specific expression of CP-AMPARs in a subset of INs and discuss the functional implications in health and disease.

## Known Roles of CP-AMPARs

Ca^2+^ is well known to play a key role in mediating synaptic plasticity (Sjöström and Nelson, 2002; Sjöström et al., 2008; Maheux et al., 2016). Consequently, CP-AMPARs are critical in regulating long-term changes in excitatory connections onto various IN types (Kullmann and Lamsa, 2007; Lamsa et al., 2007; Oren et al., 2009). Moreover, due to their voltage dependence being essentially opposite to that of NMDA receptors (NMDARs), CP-AMPARs may enable non-Hebbian plasticity at connections from PCs onto INs (Kullmann and Lamsa, 2007). For example, the induction of long-term potentiation (LTP) at excitatory inputs onto O-LM cells in the hippocampus requires presynaptic release of glutamate coincident with postsynaptic hyperpolarization, rather than the depolarization that is otherwise required for Hebbian plasticity. This non-Hebbian form of plasticity plays an important role in the hippocampal feedback circuit and may orchestrate the overall excitability of PCs (Kullmann and Lamsa, 2007). In contrast, a mechanism involving CP-AMPARs but leading to LTP of excitatory inputs onto INs in the absence of postsynaptic hyperpolarization has been observed in the basolateral amygdala (Mahanty and Sah, 1998), where excitatory synaptic transmission onto INs seems entirely meditated by CP-AMPARs. Here, tetanic stimulation leads to LTP in an NMDAR-independent yet Ca^2+^-dependent manner.

Specific IN types may also require CP-AMPARs to compartmentalize their response to excitatory inputs. In excitatory cells, dendritic spines serve as biochemical compartments, which promotes synapse specificity in long-term plasticity, which in turn ensures optimal information storage capacity (Goldberg et al., 2003; Soler-Llavina and Sabatini, 2006; Sjöström et al., 2008; Maheux et al., 2016). Although INs in general seem to have fewer spines than excitatory cells do, there appears to be a clear distinction among different classes of INs: spines are found at ∼7-fold higher density in somatostatin (Sst) than in parvalbumin (Pvalb)-expressing INs (Kawaguchi et al., 2006). Pvalb — which is highly expressed in BCs (Hof et al., 1999) — is a slow Ca^2+^-binding protein that contributes to the high endogenous Ca^2+^-buffering capacity of this cell type (Lee et al., 2000; Goldberg et al., 2003; Aponte et al., 2008). This high Ca^2+^ buffering capacity helps to compartmentalize dendritic Ca^2+^ signals in BCs without interfering with the rapid and localized CP-AMPAR-mediated Ca^2+^ transients (Goldberg et al., 2003; Aponte et al., 2008). To overcome the lack of dendritic spine-dependent Ca^2+^ compartmentalization, BCs might thus rely on the combined effects of CP-AMPARs fast kinetics and Pvalb expression. Consistent with the view that spines are particularly associated with a need for compartmentalization of relatively slow Ca^2+^ transients mediated by NMDARs, recent findings show that NMDARs are enriched in synapses onto spines as compared to dendrites in Pvalb-positive INs of mouse visual cortex (Sancho and Bloodgood, 2018 Cell Reports). Perhaps this differential localization of NMDARs and AMPARs also ties it in with their engagement in different signaling pathways. Even so, the more rapid kinetics of CP-AMPARs may ensure that compartmentalization by spines is not necessary.

## CP-AMPARs are Located at Specific Synapse Types

**FIGURE 1:**
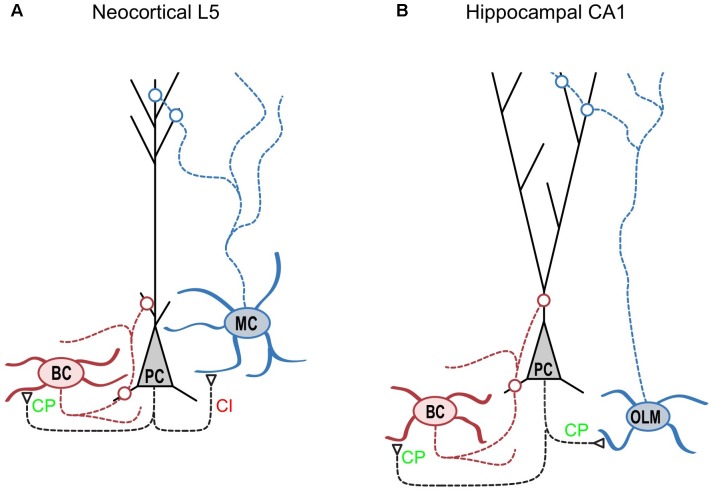
CP-AMPARs are synapse-type-specifically expressed in cortical layer 5. **(A)** As indicated by CP in green, CP-AMPARs are expressed at synapses from neocortical layer-5 pyramidal cells (“PC,” black) onto basket cells (“BC,” red) but not onto Martinotti cells (“MC,” blue) as indicated by CI in red (see Lalanne et al., 2016). Because CP-AMPARs have faster kinetics, this differential expression helps ensure that BC-mediated somatic inhibition of PCs is fast. However, fast CP-AMPARs would counteract the delayed-onset, dendritic MC-mediated feedback inhibition of PCs (Silberberg and Markram, 2007; Buchanan et al., 2012), which may help explain this differential CP-AMPAR expression. Interestingly, this expression pattern is the precise opposite to that of presynaptic NMDA receptors (Buchanan et al., 2012). **(B)** In the hippocampal CA1 circuit, CP-AMPARs are found at excitatory connections onto both BCs and O-LM cells. Interestingly, Szabo et al. (2012) also identified CP-AMPARs at PC connections onto nitric oxide synthase-expressing hippocampal INs but not onto cholecystokinin-expressing INs. Excitatory synapses are indicated by open triangles, while open circles denote inhibitory synapses. Modified from (Blackman et al., 2013) with appropriate permission.

MCs and BCs, two well-studied IN classes, are likely to take on distinctive roles in the neocortical microcircuit. While strongly facilitating excitatory inputs onto the distal dendrite-targeting MCs make them operate as high-pass filters, enabling delayed-onset feedback inhibition (Silberberg and Markram, 2007), the depressing excitatory inputs onto soma-targeting BCs make them act as low-pass filters (Blackman et al., 2013), providing early onset feed-forward inhibition of PCs (Kawaguchi and Kubota, 1997; Buchanan et al., 2012; **Figure 1**). We found that the specific expression of CP-AMPARs at PC to BC connections contributes significantly to the rapid feed-forward inhibition onto PCs (Lalanne et al., 2016), resulting in a shortened integrative time window for excitation (Pouille and Scanziani, 2001; Mittmann et al., 2005).

**FIGURE 2:**
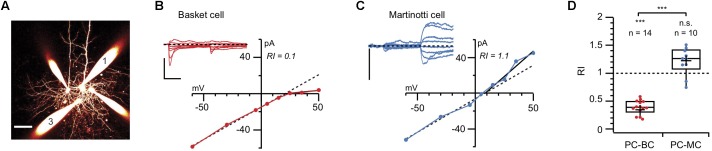
Paired recordings reveal AMPAR rectification at PC-BC but not PC-MC synapses **(A)** 2-photon microscopy maximum intensity projection of a quadruple recording where a connection from morphologically identified cell 1 (PC) to cell 3 (BC) was measured. Scale bar: 50 μm. **(B)** AMPAR responses to PC 1 stimulation in BC 3 were recorded at various holding potentials (from –60 to + 60 mV). Inward rectification in the presence of intracellular spermine indicated the presence of CP-AMPARs. Scale bars: 10 ms, 100 pA. **(C)** In another paired recording, the AMPAR currents recorded from an MC in response to stimulation of the presynaptic PC did not show inward rectification, suggesting the absence of CP-AMPARs. Scale bars: 50 pA. **(D)** The cell-specific difference in rectification was robust, as shown by the pooled values of rectification index (RI) for PC-BC (red) and PC-MC (blue) paired recordings. Modified from (Lalanne et al., 2016) with appropriate permissions.

Using a combination of immunolabelling, paired recording, AMPA uncaging, and pharmacology, we demonstrated expression of CP-AMPARs at excitatory inputs onto BCs but not MCs (Lalanne et al., 2016). Immunolabelling showed that GluA2 was almost absent from the somata of Pvalb-expressing cells, contrasting with its strong presence in the somata of Sst-positive INs and even stronger labeling in PCs. We confirmed this observation using paired recordings of connections between PCs and both IN types: unlike those in MCs, AMPAR-mediated currents in BCs were inwardly rectifying (as demonstrated by their current-voltage relationships) and were sensitive to polyamines (**Figure 2**; Lalanne et al., 2016). The synapse-specific expression of CP-AMPARs was further supported by the faster decay kinetics of currents in BCs compared to MCs.

The specific expression of CP-AMPARs in BCs, or at least in INs that express Pvalb or exhibit fast-spiking (two key properties of BCs), has been observed in several studies conducted in both neocortical and hippocampal circuits. In the rat, outside-out somatic patches from both BCs in dentate gyrus and fast-spiking INs in the neocortex showed inwardly rectifying currents with fast kinetics (Geiger et al., 1995; Koh et al., 1995b; Angulo et al., 1997). When measured, a low abundance of GluA2 mRNA correlated with these markers of CP-AMPARs expression (Geiger et al., 1995; Angulo et al., 1997). Wang and Gao (2010) also observed inward rectification of excitatory inputs onto most fast-spiking INs in the rat prefrontal cortex, presumably reflecting the expression of CP-AMPARs (also see Tao et al., 2013). Most studies of different brain areas have thus identified CP-AMPARs in BCs or at least in INs showing key BC-like properties.

In the hippocampus however, there is also convincing evidence that developmental origin rather than IN type alone determines whether or not CP-AMPARs are expressed (Matta et al., 2013). Here, medial ganglionic eminence derived IN synapses are dominated by GluA2-lacking AMPARs, whereas caudal ganglionic eminence derived IN synapses had GluA2-containing AMPARs (Matta et al., 2013) (also see Pelkey et al., 2017).

Another notable exception is the *oriens-lacunosum moleculare* (O-LM) inhibitory cell type of the hippocampus, which is not fast-spiking yet has been shown to express functional CP-AMPARs (**Figure 1B**). Although the O-LM cell type is typically thought of as the functional hippocampal homologue of the neocortical MC, there are considerable differences when it comes to molecular markers (Pelkey et al., 2017). For example, O-LM cells comprise only about 40% of Sst-expressing INs (Oliva et al., 2000; Ferraguti et al., 2004), and a subset of O-LM cells are in fact positive for Pvalb (Ferraguti et al., 2004; Chittajallu et al., 2013). In neocortex however, Sst chiefly labels MCs, and Pvalb and Sst labels are essentially mutually exclusive (Toledo-Rodriguez et al., 2005; Pelkey et al., 2017). That hippocampal O-LM cells but not neocortical MCs express CP-AMPARs widely may in other words be yet another difference in terms of molecular properties. The functional implications of this difference between MCs and O-LM cells remain unclear.

Interestingly, high-frequency stimulation was not sufficient to induce LTP at excitatory inputs onto O-LM cells, while a concomitant hyperpolarization of the postsynaptic IN was required for potentiation (Oren et al., 2009). Ca^2+^ entry at the synapse was presumably mediated by CP-AMPARs due to the alleviation of the voltage-dependent polyamine block. In contrast, studies in BCs have shown that LTP of excitatory inputs, while also mediated by CP-AMPARs, did not require postsynaptic hyperpolarization (Mahanty and Sah, 1998; Camiré and Topolnik, 2014). Interestingly, Camiré and Topolnik (2014) demonstrated the involvement of internal Ca^2+^ stores in mediating LTP in the absence of postsynaptic hyperpolarization. Because this mechanism was not identified in O-LM cells, CP-AMPARs may thus play a role in mediating LTP in different cell types via distinct signaling pathways.

The emerging picture is not black-and-white, but quite nuanced. In some brain regions and at certain developmental stages, CP-AMPAR expression goes with cell and synapse type (Lalanne et al., 2016; **Figures 1A**, **2**), but this may not hold true in other brain regions (Szabo et al., 2012; **Figure 1B**). Yet again, at other time points, factors such as developmental origin may play a relatively more dominant role in determining CP-AMPAR expression patterns (Matta et al., 2013).

## Functional Implications in Health and Disease

The regulation of CP-AMPAR expression implies specific functional relevance, which in turn suggests that CP-AMPAR dysregulation is involved in pathology. Here below, we briefly discuss the functional role of CP-AMPARs in health and disease.

CP-AMPARs have been implicated in controlling synaptic short-term dynamics. Lu et al. (2014) reported the presence of CP-AMPARs at local but not at long-range inputs to Pvalb-positive INs in L2/3 of the mouse visual cortex. Interestingly, they observed a selective developmental decrease in short-term depression of these local inputs, which correlated with a developmentally increased ratio of CP- to CI-AMPARs. This was supported by rectifying current-voltage relationships and a higher sensitivity to the CP-AMPAR blocker 1-naphthyl acetyl spermine (NASPM) at mature (postnatal day 32–34) short-range excitatory synapses onto Pvalb-positive INs, in comparison to linear current-voltage relationships and less sensitivity to NASPM at both short- and long-range synapses in younger animals (postnatal day 17–19). Furthermore, blocking CP-AMPARs strongly affected the short-term dynamics of mature but not juvenile short-range connections, by rendering them more depressing. During high-frequency stimulation, CP-AMPARs are enhanced by use-dependent relief from polyamine block (Rozov and Burnashev, 1999; Rozov et al., 2001). Polyamine-dependent facilitation of CP-AMPARs thus counteracts short-term depression at excitatory synapses. This explains how blockade of postsynaptic CP-AMPARs can somewhat counterintuitively increase short-term depression, which is typically attributed to presynaptic mechanisms (Abbott and Regehr, 2004; Blackman et al., 2013).

One intriguing aspect of the study by Lu et al. (2014) – which echoes the findings of Toth and McBain (1998) in the hippocampus – is the suggestion that different inputs onto a single Pvalb IN can activate different subtypes of AMPARs. This notion, that plasticity is regulated at the synaptic level, has been termed synapse-type-specific plasticity (STSP) (Larsen and Sjöström, 2015), and may pertain to short as well as long-term plasticity (Blackman et al., 2013). STSP should thus not be confused with synapse specificity in long-term plasticity, which maximizes information storage by preventing spread of connective strengthening or weakening to neighboring synapses (Goldberg et al., 2003; Soler-Llavina and Sabatini, 2006; Sjöström et al., 2008; Maheux et al., 2016). The synapse-type-specific developmental decrease of short-term depression observed by Lu et al. (2014) may reflect a key role of CP-AMPARs in neuronal maturation via STSP (Larsen and Sjöström, 2015).

In our study (Lalanne et al., 2016), all experiments were carried out using tissue from mice aged from P14-P21, a relatively narrow age range. In many cell types, the expression of CP-AMPARs has been found to vary with development, as determined by immunolabelling, electrophysiology and/or pharmacology. Unfortunately, no clear-cut universal pattern is apparent: different types of neurons and brain regions have different developmental profiles. For example, several studies have demonstrated a developmental decrease in the expression of CP-AMPARs (Kumar et al., 2002; Shin et al., 2005; Osswald et al., 2007; Soto et al., 2007; Lu et al., 2014) while one has shown expression to fluctuate with age (Wang and Gao, 2010). Of greatest relevance to our study, in Pvalb-positive INs of the mouse visual cortex the expression of CP-AMPARs has been shown to be elevated at P31–P34 when compared to that at P17–P19 (Lu et al., 2014). Clearly, further studies are required to resolve the developmental regulation of CP-AMPAR expression.

As mentioned earlier, CP-AMPAR developmental regulation suggests a possible contribution to pathology when dysregulated. Indeed, this receptor type has long been suggested to play crucial roles in excitotoxicity and cell death (for reviews, see Liu and Zukin, 2007; Wright and Vissel, 2012; Henley and Wilkinson, 2016). In particular, an increased expression of CP-AMPARs following neurological insult may enhance glutamate toxicity due to elevated Ca^2+^ influx, a concept known as the GluA2 hypothesis (Pellegrini-Giampietro et al., 1997). For example, following seizures of various types, GluA2 expression is decreased (Prince et al., 2000; Rajasekaran et al., 2012; Lorgen et al., 2017). Although lowered GluA2 expression does not necessarily in itself lead to cell death (Wiltgen et al., 2010), an increase of CP-AMPAR-dependent Ca^2+^ influx was shown in CA1 hippocampal neurons following hypoxia-induced neonatal seizures (Lippman-Bell et al., 2016). Antagonizing AMPARs with NBQX after *in-vivo* hypoxia prevented both expression of GluA2-lacking AMPARs and the enhanced Ca^2+^ influx (Lippman-Bell et al., 2016). Interestingly, post-hypoxia induction of CP-AMPAR expression correlated with an impairment of LTD, which was restored by *in-vivo* administration of NBQX (Lippman-Bell et al., 2016). Since LTD requires relatively low Ca^2+^ influx, this result is consistent with excess Ca^2+^ influx caused by the increased CP-AMPARs expression following hypoxia. By sequentially blocking CP-AMPARs, NMDARs or L-type Ca^2+^ channels, the authors also demonstrated that the excessive Ca^2+^ influx is primarily due to the expression of CP-AMPARs.

## Conclusion and Future Directions

As reviewed above, our work revealed that synapse-specific CP-AMPAR expression at PC-to-BC connections (**Figure 1**) helps to further speed up BC inhibition, because of the rapid kinetics of the CP-AMPAR-mediated currents (Lalanne et al., 2016). This implies that CP-AMPARs not only mediate synaptic plasticity (Kullmann and Lamsa, 2007), but are also important for proper information transfer across synapses. This conclusion echoes that reached concerning NMDARs, which are well-known for their role in mediating Hebbian plasticity and memory formation (Sjöström and Nelson, 2002; Sjöström et al., 2008; Nabavi et al., 2014; Maheux et al., 2016), but which also play an important role in neurotransmission and in functional computations of neocortical microcircuits (Salt, 1986; Schiller et al., 2000; Lavzin et al., 2012). Interestingly, we found that unlike CP-AMPARs, postsynaptic NMDARs were relatively uniformly and not synapse-specifically expressed in neocortical layer 5 (Lalanne et al., 2016). In contrast, a study by Le Roux et al. (2013) revealed a synapse-type-specific expression of postsynaptic NMDARs: while excitatory feed-forward connections onto Pvalb-expressing CA1 INs expressed few NMDARs, feed-back connections onto the same INs expressed high levels of NMDARs, perhaps indicative of a difference between neocortex and hippocampus. On the other hand, *presynaptic* NMDARs (preNMDARs) are expressed in a synapse-specific manner in neocortical circuits, opposite and complementary to that of CP-AMPARs, so that PC-MC but not PC-BC connections possess preNMDARs (Buchanan et al., 2012). This arrangement makes good sense, since preNMDARs help wind up PC-MC excitatory connections during sustained high-frequency firing by boosting the replenishment of the readily releasable pool of vesicles (Abrahamsson et al., 2017). A functional image of differential inhibitory signaling emerges: while preNMDARs at PC-MC synapses help enable late-onset and sustained inhibition of PCs, CP-AMPARs at PC-BC connections promote brief, rapid-onset inhibition of PCs.

The notion that synaptic properties such as long- and short-term plasticity depend on synapse type, STSP, has gained increasing interest in recent years (Blackman et al., 2013; Larsen and Sjöström, 2015; Nusser, 2018). This idea, however, has been around since the 1970s, when, for example, it was shown that synapses of the same axon but with differential release properties innervated different muscles (Parnas, 1972). STSP has subsequently been reported in hippocampus (Scanziani et al., 1998; Toth et al., 2000; Pouille and Scanziani, 2004; Sylwestrak and Ghosh, 2012; Neubrandt et al., 2018), cerebellum (Bao et al., 2010), and neocortex (Thomson, 1997; Markram et al., 1998; Reyes et al., 1998). As a general principle, soma-targeting BCs receive short-term depressing excitatory drive, while dendrite-targeting MC-like INs receive facilitating excitatory inputs (Blackman et al., 2013). This differential arrangement of short-term plasticity separates early- and late-onset inhibition onto soma and dendrites, respectively, of PCs (Pouille and Scanziani, 2004). Future research may reveal how synapse-type-specific expression and functioning of receptors such as CP-AMPARs (Lalanne et al., 2016) and preNMDARs (Buchanan et al., 2012) or down-stream signaling proteins such as RIM1 or JNK2 (Abrahamsson et al., 2017) control STSP.

The synapse-type-specific expression of CP-AMPARs found in neocortical microcircuits (Lalanne et al., 2016) appears to generalize to other brain regions such as hippocampus (Lamsa et al., 2007; Nissen et al., 2010), striatum (Gittis et al., 2010; Gittis et al., 2011) and cerebellum (Soler-Llavina and Sabatini, 2006), but additional work is needed to determine if this holds true at all developmental time points. Although a link between CP-AMPARs and specific forms of long-term plasticity has been quite firmly established (Kullmann and Lamsa, 2007; Lamsa et al., 2007; Nissen et al., 2010; Szabo et al., 2012), it will be important to understand how synapse-type-specific CP-AMPAR expression impacts circuit remodeling, as well as what the functional consequences are, in health as well as in disease. In particular, the association of synapse-type-specific CP-AMPAR expression with epilepsy and excitotoxicity deserves further exploration. Although the GluA2 hypothesis classically refers to the involvement in disease of principal cells (Pellegrini-Giampietro et al., 1997), there is no *a priori* reason to assume that this hypothesis cannot apply to INs. A link between STSP, CP-AMPARs in INs, and pathology thus beckons.

## Author Contributions

All authors listed have made a substantial, direct and intellectual contribution to the work, and approved it for publication.

## Conflict of Interest Statement

The authors declare that the research was conducted in the absence of any commercial or financial relationships that could be construed as a potential conflict of interest.
